# Analysis of factors influencing community participation in the administration of vaccination activities in the health district of Tône in Togo in 2024

**DOI:** 10.4102/jphia.v17i1.1925

**Published:** 2026-10-28

**Authors:** Ahmed Kabore, Goumban Bangoure, Relwende A. Yameogo, Grâce-Alice E.B. Gumedzoe-Hoseto, Rayendé J.R. Soubyabiga, Akossito H. Tognon, Boureima Coulibaly, Baba Kalle, Ahmat K. Abdelhakim, Nestor Bationo, Lorraine M.P. Bougma, Nicolas Meda

**Affiliations:** 1 Department of Public Health, Health Sciences Research and Training Unit, Joseph KI-ZERBO University, Ouagadougou, Burkina Faso

**Keywords:** barrier, community participation, vaccination, child, Tône, Togo

## Abstract

**Background:**

Limited community involvement remains a major challenge to achieving full immunisation coverage in sub-Saharan Africa. In Togo, despite multiple strategic efforts under the Expanded Programme on Immunization (EPI), vaccination coverage remains suboptimal, with a growing proportion of zero-dose children.

**Aim:**

This study aimed to analyse the barriers to community involvement in childhood immunisation activities in the Tône health district of Togo in 2024.

**Setting:**

The study was conducted in the Tône health district, located in northern Togo, characterised by both rural and semi-urban settings with heterogeneous geographic accessibility to health facilities.

**Methods:**

A qualitative exploratory study with a cross-sectional design was conducted from November 2023 to September 2024. A purposive sampling strategy was used to recruit 112 participants, including parents, community health workers, religious and traditional leaders, health officials and local authorities. Data were collected through individual interviews and focus group discussions and analysed thematically using NVivo14.

**Results:**

Barriers were grouped into three main domains: (1) *administration of immunisation services* (ignorance, poor reception, fear of adverse events following immunisation, vaccine stock-outs, long waiting times, distance to facilities and traditional practices); (2) *social mobilisation* (low awareness, lack of incentives, social media influence and limited collaboration); and (3) *community financing* (poverty, low income and transport costs).

**Conclusion:**

Community involvement in EPI remains constrained by intertwined sociocultural, institutional and economic barriers.

**Contribution:**

This study provides contextual evidence to inform targeted communication strategies and community-based interventions aimed at improving immunisation uptake in fragile settings.

## Introduction

The Global Immunization Action Plan considers that a lack of community involvement is a major obstacle to full immunisation of children.^1^ This involvement is an essential component for the success of primary healthcare (PHC) application.^2^ It is a new initiative for the Global Alliance of Vaccine and Immunisation (GAVI) to increase vaccination coverage.^3^ The insufficient implementation of this strategy has a negative impact on vaccination programmes.^4^

In 2020, worldwide, 22.7 million children (17% of the target) did not receive a third dose of diphtheria, tetanus and pertussis (DTP3), compared to 19 million (14%) in 2019. Children who did not receive the first dose of diphtheria, tetanus and pertussis (DPT) at 12 months (zero-dose children) were approximately 17.1 million in 2020.^5^ Indeed, the failure of vaccination programmes with low immunisation coverage results in the occurrence of vaccine-preventable diseases (VPD).^6^

In Africa, WHO states that community involvement is a perspective for successful immunisation.^7^ About 12.7 million children were under-vaccinated in 2021, of which 8.7 million were ‘zero-dose’ children, largely due to disruptions caused by the COVID-19 pandemic, which reversed nearly a decade of progress in routine childhood immunisation.^8^

In Togo, the active participation of the community is one of the nine guiding principles of primary healthcare (PHC).^9^ Low community involvement is the basis for the failure of health programmes and the failure to achieve their objectives.^4^ The proportion of zero-dose children increased from 5.1% in 2017 to 12% in 2021.^10,11^ This increase in zero-dose children could be the basis of the epidemics related to Maladies évitables par la vaccination (EVM) [Vaccine-Preventable Diseases {VPDs}] (meningitis, measles, rubella, poliomyelitis, etc.) that the country experiences every year.^10^ Studies conducted in Togo have identified several factors influencing vaccination coverage among children. In 2018, a study reported that low community involvement in Expanded Programme on Immunization (EPI) implementation was one of the key factors for zero-dose children.^12^ In addition, the level of education of mothers, the economic situation of the household, the distance of households to health training and the lack of time of parents are also associated with the low vaccination coverage in the country.^13^

Several actions are implemented to strengthen community involvement in EPI activities.^14^ The Ministry of Health, through the Immunization Division DI to ID for Immunization Division (DI), has developed the comprehensive multi-year plan for the 2016–2020 ENP. It aims to ensure access and use of vaccination services.^15^ Other strategic documents are developed, such as the integrated communication plan of the EPI^16^ and the national vaccination strategy 2023–2026.^17^ Furthermore, the National Health Development Plan and the Public Health Code of Togo recommend community involvement and accountability in the organisation, planning, execution and evaluation of health programmes and/or projects.^14,18^ In addition, the DI to ID for Immunization Division DI through the Civil Society Organizations Platforms for Vaccination and Immunization (POSCVI) has implemented strategies such as awareness campaigns and educational talks, the search for lost children, the celebration of Child Health Days, the training of community actors, the mobilisation of proximity, community dialogues and the establishment and revitalisation of ‘champions dads’ initiative^19^ on the participation of men in the vaccination of children from 0 years to 5 years old.

Despite these actions, the EPI of Togo continues to face low community involvement, resulting in low vaccination coverage. In Togo, few studies have addressed the factors that influence community involvement in EPI activities.^5^ In this perspective, we explored the barriers to community involvement in the administration of the EPI, the barriers to community mobilisation and the barriers to financing vaccination activities.

## Research methods and design

### Study design

It was a qualitative exploratory study using a cross-sectional design conducted between November 2023 and September 2024.

### Setting

The study was carried out in the sanitary district of Tône, located in northern Togo. The district includes public and private health facilities that provide immunisation services and spans both urban and rural settings with varying levels of geographic accessibility. Villages are unevenly distributed in relation to health facilities, with some located less than five kilometres away and others situated beyond this distance. This diversity in accessibility was considered relevant to understanding variations in community engagement in immunisation activities.

### Study population and sampling strategy

The study population focused on individuals, community organisations and institutional actors who could influence participation in child immunisation activities. Participants included community health workers (CHWs), parents of children aged 0–23 months, traditional leaders, religious leaders, health facility officers (HFO), members of Health Management Committees (HMC), women’s associations (Mothers’ Clubs), the Prefectural Director of Health (PDH), the District Health Promotion Officer (DHPO) and mayors.

A purposive sampling strategy was used to select both study participants and study sites based on their potential to provide rich and relevant information regarding the factors influencing the phenomenon under investigation. Villages and districts attached to selected health facilities were identified through purposive selection to capture contrasting contexts. Specifically, villages located less than five kilometres from health facilities and those located more than five kilometres away were included to explore potential differences related to geographic access. Participants were purposively recruited because of their direct experience, knowledge or involvement in the study topic, including parents, community health workers, religious and traditional leaders, health officials and local authorities.

The sample size was determined by the principle of data saturation. Recruitment was discontinued when additional interviews and focus group discussions no longer generated new relevant information.

### Data collection

Data were collected using two qualitative techniques: semi-structured in-depth interviews (IDIs) and focus group discussions (FGDs). Semi-structured interviews were conducted using an interview guide and involved parents, traditional leaders, religious leaders, health officers, district authorities and mayors. Focus group discussions involved CHWs, Mothers’ Clubs members and HMC members, using a dedicated discussion guide.

Data were collected by the principal investigator and two trained research assistants with prior experience in qualitative data collection. Before fieldwork, the research team received additional training on qualitative interviewing techniques, focus group facilitation, research ethics and note-taking procedures. Semi-structured in-depth interviews and focus group discussions were conducted using interview guides developed from the study objectives.

The focus group discussions were moderated by an experienced qualitative researcher, while a second researcher took detailed field notes and managed the audio recording. Data collection took place in private locations within the communities or health facilities to ensure confidentiality and participant comfort. Interviews and discussions were conducted in French and the local language (Moba), according to participants’ preferences. When local languages were used, recordings were translated and transcribed into French for analysis.

The in-depth interviews lasted approximately 30 min, while focus group discussions lasted between 60 min and 90 min. All interviews and discussions were audio-recorded with participants’ permission, and field notes were taken to capture contextual information and non-verbal interactions.

### Data analysis

Data were analysed using thematic analysis supported by NVivo 14 software. All interviews and focus group discussions were transcribed verbatim. The transcription process was supported by a master’s-level public health student and subsequently verified for accuracy against the audio recordings. The research team reviewed the transcripts repeatedly to familiarise themselves with the data and identify emerging patterns. An inductive coding framework was developed based on themes emerging from the data. Codes were organised hierarchically into parent (father) and child codes to reflect thematic relationships and were subsequently grouped into broader categories and themes.

NVivo 14 software was used to manage, organise and code the data. To enhance the rigour and reliability of the analysis, transcripts were independently coded by two researchers. The coded datasets were compared and merged, resulting in an inter-coder agreement rate of 99.26%. A third investigator acted as an arbiter in cases of coding discrepancies and contributed to reaching consensus on code application and theme development. The use of multiple coders, transcript verification and investigator triangulation strengthened the credibility, dependability and confirmability of the findings.

All interviews and focus group discussions were transcribed verbatim. The transcription process was supported by a master’s-level public health student and subsequently verified for accuracy. A coding framework was developed based on themes emerging during data collection. Codes were organised hierarchically into father codes and child codes to reflect thematic relationships.

NVivo 14 software was used to import and code the transcribed data. To enhance analytical rigour, coded data were merged, and an inter-coder agreement rate of 99.26% was calculated. A third investigator acted as an arbiter in cases of discrepancy during the calculation of the match rate, thereby strengthening the reliability of the analysis.

### Ethical considerations

The study protocol and interview guide were submitted to the Committee of Bioethics for Health Research of Togo (CBRS). Ethical clearance to conduct this study was obtained from the Ministry of Health and Public Hygiene Research Ethics Committee (No. 011/2024/CBRS) on 16 May 2024, after which the process was continued by obtaining the collection authorisation from the Minister of Health and Public Hygiene of Togo. The collection was made after informed, voluntary and free consent of the participants. Each participant benefited from an explanation to better understand the subject of study. A written consent form was offered to participants for individual interviews. In addition, all original interview recordings and transcripts of verbatim records were securely stored on an external hard drive for a period of 12 months, from the end of the investigation (27 July 2024). The confidentiality of the participants in the study was ensured through the use of verbatims. To preserve anonymity in the transcription, participants were coded as follows: Nom_Number. The name corresponded to ‘Parents of children’, ‘traditional leader’, ‘religious leader’, ‘HFO’, ‘PDH’, ‘DHPO’, ‘mayor’ for individual interviews and ‘CHWs’, ‘HMC’, ‘Mothers’ Club’ for the focus group. The numbers corresponded to the order of the interview for individual interviews and the speaking order for focus groups.

## Results

### Socio-demographic characteristics

A total of 112 participants, including 60 women, were included in the study. Data were collected through seven focus groups (*n* = 67) and 45 individual interviews. The community health workers (*n* = 35, including 16 women) were organised into three focus groups, with one focus group conducted per health facility. Thirty parents of children aged 0 months to 23 months participated in the study, including five fathers (16.7%) and 25 mothers, from six villages located both within and beyond 5 km of health facilities. Individual interviews were conducted with five religious leaders, three traditional chiefs, two mayors, three health formation managers, the district health promotion manager and the Prefectural Director of Health. Members of the Health Management Committees (HMC) participated in two health facilities in the Tône district, with a total of five participants. Finally, 27 members of the Mothers’ Club participated in two focus groups comprising 12 and 15 participants, respectively.

### Barriers to community involvement in the expanded programme on immunisation

The thematic analysis identified barriers to community involvement across three main domains: EPI administration, social mobilisation and community financing. Within these domains, several themes and sub-themes emerged from the data.

### Barriers to community involvement in expanded programme on immunisation administration

Thematic analysis identified several themes and sub-themes describing barriers to community involvement in EPI administration. These findings highlight the influence of sociocultural, health service and accessibility barriers on participation in routine immunisation. Representative quotations are presented in Table 1.

**TABLE 1:** Thematic synthesis of barriers to community involvement in expanded programme on immunisation administration.

Theme	Sub-theme	Illustrative codes	Verbatim
Sociocultural barriers	Knowledge deficit and vaccine hesitancy	Ignorance False rumours about vaccines	‘In my opinion, I can also say that there is ignorance among women; there are many women who think that by not vaccinating their children will not cause any illness in the child.’ (Focus Group CHWs 3) ‘… often people are poorly informed especially at the moment there is false information that advises some moms not to take their children to be vaccinated as it can still cause them illnesses.’ (Religious leader 2) ‘There are these cases, some of whom refuse to say that her child is other well but when she went to vaccinate her child the next day, the child was not sick earlier, the child has now fallen ill. There is a fever that happened to her child. There are these cases-there those women who now refuse to the next vaccination they no longer want to bring their children.’ (Traditional leader 1)
		Fear of AEFI lack of trust	
	Traditional Beliefs	Traditional practices	‘There are also others who say that they have opted for the tradition of herbal teas, it’s them other than their former parents’ time they processed the herbal teas until they grew up to even older than today when it is now our vaccines-there that kills us quickly we can’t reach our ancestors so they also want to opt for these kinds of practices so it’s also what makes people refuse vaccines.’ (Focus Group CHWs 3: Participant 2)
Health service barriers	Service quality	Bad reception Patient discrimination	‘Sometimes we also go to the vaccination, the agents who vaccinate us there they scold the friends that they don’t like it sometimes they will take the notebooks thrown on the ground you will go pick that up too it doesn’t encourage us to go to the vaccination.’ (Parent 6) ‘Yes, if you are interested, there are also some health workers, especially those who are passive. excuse the term because you are a Muslim. I am a Christian; they come to me first because I belong to their religion, it’s an insufficiency.’ (Religious leader 4) ‘That can never motivate her because when she came very early, she thinks we will receive her and then she will quickly leave maybe-to be by coming she has no money at least to find something to eat she expects others as soon as she is there we will receive her quickly and then she will leave when time really goes away time goes away like that she is discouraged.’ (Traditional leader 1)
		Social inequality Long waiting time	
Accessibility barriers	Geographic accessibility	Natural obstacle	‘Houn can be a barrier, for example, another village on the other side there is a large backwater that separates us, there is a large backwater. Sometimes the woman wants to vaccinate her child; now the backwater is full, she cannot come, and she too, since there is a backwater, good, she does not come. More.’ (Parent 14)

AEFI, adverse events following immunisation; CHW, community health worker.

### Barriers to community involvement in social mobilisation for the expanded programme on immunisation

Barriers to community involvement in social mobilisation were organised into four major themes: communication, capacity, governance and participation barriers. Representative quotations illustrating these themes are presented in Table 2.

**TABLE 2:** Thematic synthesis of barriers to community involvement in social mobilisation for the expanded programme on immunisation.

Theme	Sub-theme	Illustrative codes	Verbatim
Communication barriers	Information and awareness	Low community awareness	‘Well, I think that already heè it is the lack of information at the community level that can do because to make a social mobilisation you still need to master the subject in order to be able to give the real information it may be the main obstacle the lack of information of the communities to be able to do this mobilization-there.’ (PDH) ‘Yes, because we talk, they come, some staff are sent to us here; they don’t understand Moba, so our moms or our dads also don’t understand French, so now they speak, so someone is needed to interpret, and if someone misinterprets, it can also be a cause.’ (HMC Focus Group 2: Participant 1) ‘Today, we also need to translate technical terms from medicine into our mother tongues; sometimes they are not well explained in the mother tongue. The language barrier is there, so today we need to provide information in our mother tongues.’ (Religious leader 2)
		Language barrier	
Capacity barriers	Skills and knowledge	Lack of training for community leaders and CHWs	‘It is due to a lack of training that they are not trained: the Village Development Committees, the Neighborhood Development Committees, are not trained, even there are the Cantonal Development Committees, which are not trained if they were involved in health training. the importance of the thing they are not trained.’ (Traditional leader 2)
		Low level of education	‘There is also that the one who has been to school and the one who has not been to school it cannot be, even that the one who has been to school knows the merits of vaccination the one who has not been to school vaccinated because he must be vaccinated but he does not know why he is going to vaccinate his child.’ (Parent 27)
Governance barriers	Community structures	Dysfunction of community associations	‘[*I*]t should be the community that should help us to raise awareness among these kinds of people, especially HMC, but HMC frankly does not get too involved in vaccination activities, we must say so, we must not hide HMC is not involved in vaccination I believe that no more than a month in supervision in the health district it has been revealed that there is a low involvement of HMC in the context of health activities, so for them their activities stop at the level of management of generic essential drug (MEG) and funds or not only the management of health structures as such but should also contribute to mobilising the population for the causes of health.’ (PDH) ‘The second element, I think there is a lack of dialogue with the communities, I will talk about there are what they call interviews, these are community-level interviews and the commitment to vaccination. They often hold meetings with communities to discuss these obstacles so that the community itself can propose approaches to solutions.’ (PDH)
		Lack of collaboration with the community	
Governance barriers	Political influences	Political influence of the CHWs choice	‘I wanted to add something since I am here and during our monthly meetings we find these problems-Elsewhere, an CHW who was chosen by his brother or his uncle is Kombonnaba, whereas the population doesn’t like him at first; he’s a drunk, he gets drunk, he won’t even do outreach or vaccination, so that encroaches on the health, what we call the vaccination of children is a problem in the neighborhood, thank you.’ (Focus Group CHW 3: Participant 2) ‘Compared to HMC, it’s the same for others who want it to be their family and others who want to impose as it is, it has to be that one.’ (HFO 2)
		Choice of HMC by political affiliation	
Participation barriers	Opportunity costs	Occupation of community members	‘If I take as an example the case of awareness-raising, leaving one place to go to another to listen to awareness-raising, people see that it’s a waste of time. I have to spend time listening to awareness-raising, against what is it compared to my field that awaits me at my market? What awaits me in relation to many things there are many factors that we look at.’ (Religious leader 5) ‘Heeéh as they say money is the sinister of war it’s real we will not deny that if we charge people for certain services without remuneration as they say it can happen that people may neglect certain things when they do not feel supported.’ (Religious leader 3)

CHW, community health worker; PDH, Prefectural Health Director; HMC, Health Management Committee; HFO, Health Facilitator Officer.

### Barriers related to community financing of expanded programme on immunisation activities

Economic barriers emerged as the main factors limiting community involvement in financing EPI activities. Two sub-themes household poverty and transportation constraints, summarise participants’ experiences. Representative quotations are presented in Table 3.

**TABLE 3:** Thematic synthesis of barriers to community financing of expanded programme on immunisation activities.

Theme	Sub-theme	Illustrative codes	Verbatim
Economic barriers	Household poverty	Poverty	‘I can say all that there is poverty there is poverty if we say that there is no poverty we lie we are in the middle of town but people can’t even eat twice a day there is poverty sincerely.’ (Focus Group CHWs 1) ‘That’s it, we must not be ignored; there is a somewhat low standard of living, people are looking for themselves so to eat, travel is quite an investment. Indeed, there is this situation which hinders many activities such as vaccination. They have the priority and we ask them to pay for this. here it’s not easy.’ (Religious leader 1)
		Low income level	
Economic barriers	Transportation constraints	Transport costs	‘We don’t have the means if the house is far from the centre, the woman has to leave until she goes to the centre to get vaccinated, the child; you, the man also, don’t have a motorcycle. How are you going to accompany the lady? It’s a bit difficult if the lady sees that she is late, good. she is obliged to leave.’ (Focus Group CHWs 2: Participant 6) ‘It suffices that a woman who is more than five kilometres away must leave to go vaccinate her child, it will still cost people have no way to finance these transports in these remote villages, here someone who is going to leave Worgou to come to the Polyclinic here if they are taken seven hundred francs to and fro one thousand four hundred francs it can be a hindrance for this lady to come to the health structure.’ (PDH)

CHW, community health worker; PDH, Prefectural Health Director.

### Similarities and differences in perceived barriers across participant groups

A comparative analysis across participant groups revealed substantial convergence in the perception of the major themes and sub-themes, although some differences emerged according to participants’ institutional roles and community responsibilities. These similarities and differences are summarised in Table 4.

**TABLE 4:** Similarities and differences of barriers to community involvement according to the areas of the expanded programme on immunisation.

Theme	Sub-theme	Traditional leaders	PDH	Mayor	Family	HFO	Religious leaders	DHPO	HMC	CHWs	Mothers’ clubs
Sociocultural barriers	Knowledge deficit and vaccine hesitancy	✓	✓	✓	✓	✓	✓	✓	✓	✓	✓
	Traditional beliefs	-	-	✓	✓	✓	✓	✓	✓	✓	-
	Household attitudes	-	-	✓	✓	✓	-	-	-	-	-
Health service barriers	Service quality	✓	✓	✓	✓	✓	✓	✓	✓	✓	✓
	Service organisation	✓	✓	-	✓	✓	✓	✓	✓	✓	✓
Accessibility barriers	Geographic accessibility	✓	✓	✓	✓	✓	✓	✓	✓	✓	✓
Communication barriers	Information and awareness	✓	✓	✓	✓	✓	✓	✓	✓	✓	✓
Capacity barriers	Skills and knowledge	✓	✓	✓	✓	✓	✓	-	✓	✓	✓
Governance barriers	Community structures	-	✓	✓	✓	✓	✓	-	✓	✓	-
	Political influences	✓	✓	✓	✓	✓	✓	✓	✓	✓	✓
Participation barriers	Opportunity costs	✓	✓	✓	✓	✓	✓	✓	✓	✓	✓
Economic barriers	Household poverty	✓	✓	-	✓	✓	✓	-	✓	✓	✓
	Transportation constraints	-	✓	-	✓	✓	-	✓	✓	✓	✓

Note: Overall, sociocultural, health service, accessibility and communication barriers were consistently identified across participant groups. In contrast, governance- and household-related sub-themes were more frequently emphasised by specific stakeholder groups, reflecting differences in institutional responsibilities and community experiences.

HFO, Health Training Officer; PDH, Prefectural Health Director; DHPO, District Health Promotion Manager; HMC, Health Management Committee; CHW, community health worker.

## Discussion

This study explored barriers to community involvement in childhood immunisation in the Tône health district. The findings showed that these barriers are multidimensional and extend beyond individual attitudes to include health system, governance and socioeconomic factors. The thematic analysis identified three major domains of barriers: those related to EPI administration, social mobilisation and community financing. Together, these findings suggest that improving community participation in routine immunisation requires interventions that simultaneously strengthen community awareness, improve the quality of health services, reinforce community engagement mechanisms and address financial constraints.

This study explored barriers to community involvement in childhood immunisation in the Tône health district. The findings showed that these barriers are multidimensional and extend beyond individual attitudes to include health systems, governance and socioeconomic factors. The thematic analysis identified three major domains of barriers: those related to EPI administration, social mobilisation and community financing. Together, these findings suggest that improving community participation in routine immunisation requires interventions that simultaneously strengthen community awareness, improve the quality of health services, reinforce community engagement mechanisms and address financial constraints.

### Barriers to community involvement in expanded programme on immunisation administration

Barriers related to EPI administration were the most diverse, encompassing sociocultural, health service and accessibility factors. Participants highlighted limited knowledge of vaccination, fear of adverse events following immunisation (AEFI), false rumours, lack of trust in vaccines and traditional beliefs as important determinants of vaccine hesitancy. Similar findings have been reported in Nigeria, where misconceptions about childhood vaccination^20^ and traditional practices reduced confidence in conventional medicine.^21^ Low educational attainment has also been associated with limited knowledge of immunisation schedules and AEFI in Burkina Faso and Cameroon.^22,23^ In the Savanes region, where school dropout remains high,^24^ these educational disparities may further reinforce misconceptions regarding vaccination. Likewise, inadequate community awareness and ineffective communication by health workers have been identified as factors limiting caregivers’ knowledge of immunisation services.^22,25,26^ Similar findings have also been reported in India,^27^ while another study identified poor knowledge of subsequent vaccination appointments as a determinant of incomplete childhood immunisation.^28^

Participants also described several health service barriers, including poor reception by health workers, long waiting times, perceived discrimination, shortages of health personnel, vaccine stock-outs and organisational constraints related to vaccination policies. These findings are consistent with previous studies showing that inadequate staffing,^29^ insufficient provider training,^1^ and heavy workloads^30^ negatively influence the quality of provider–client interactions^31^ and discourage caregivers from returning for subsequent vaccination visits. Similar observations regarding disrespectful treatment and discrimination have been reported in Chad^32^ and Nigeria,^21^ where negative experiences^33^ during of vaccination sessions reduced community confidence in immunisation services.^8^ Fear of AEFI was further reinforced by misinformation circulating through communities and social media,^34,35^ while concerns regarding vaccine side effects have similarly been reported in Saudi Arabia and other settings as barriers to complete childhood immunisation.^28,36,37^ Finally, geographical distance and natural obstacles further limited access to vaccination services, particularly for households living in remote communities.^38^

### Barriers to community involvement in social mobilisation

The findings indicate that effective community mobilisation is constrained by weaknesses in communication, community capacity, governance and motivation. Limited community awareness, language barriers, inadequate training of community leaders and CHWs and poor collaboration between health services and communities reduced the effectiveness of awareness activities. Similar findings have been reported in India, where community health workers experienced difficulties in conducting health education because of limited training.^39^ Studies conducted in Pakistan^40^ and Somalia^41^ also identified insufficient community awareness as a major obstacle to participation in childhood immunisation activities.

Governance challenges also emerged as important barriers. Participants highlighted the dysfunction of community associations, political influence in the selection of CHWs and HMC members and limited confidence in community volunteers. Such practices may reduce community ownership of immunisation programmes and weaken trust in local health governance. In addition, lack of monetary incentives and competing livelihood priorities discouraged participation in community mobilisation activities. Similar findings have been reported in India and Pakistan,^27,40^ while a study conducted in rural Bangladesh demonstrated that the absence of incentives reduced community participation in health-related activities.^42^

### Barriers to community financing of expanded programme on immunisation activities

Economic barriers were consistently identified as important obstacles to community involvement in financing immunisation activities. Poverty, low household income, transport costs and limited access to transportation reduced caregivers’ capacity to participate in vaccination activities despite vaccines being provided free of charge. Similar associations between financial constraints and incomplete childhood immunisation have been reported in several settings.^21,38^ In the Savanes region, where poverty remains the highest in Togo,^43^ households often prioritise meeting immediate subsistence needs over preventive healthcare. Low agricultural productivity and limited economic opportunities further constrain household resources.^44,45^ Similar findings have been reported in Bangladesh^42^ and Somalia,^41^ where poverty and transport costs limited both access to vaccination services and broader community participation in health programmes.

### Strengths and limitations of the study

Our sample was selected in a reasoned manner, thus giving our results a contextual character depending on the place, the time and the actors involved. The use of a dictaphone to record interviews could cause distrust among some participants; this can lead to reluctance to share sensitive information that may be valuable for our research. Also, the advocacy aspect at the political level has not been explored and will be the subject of other studies.

## Conclusion

Our study aimed to analyse the barriers to community involvement in EPI activities. The low involvement of the community in the EPI jeopardises the achievement of its objectives, despite several years of its implementation. Our results revealed that the main obstacles were ignorance, poor reception, fear of AEFI and vaccine disruption. For social mobilisation in favour of the EPI, the lack of monetary incentives, the low awareness of the community, and the occupations of the community members were the obstacles. In addition, low income and poverty were factors limiting community financing of EPI activities.

These results facilitate the formulation of appropriate communication strategies aimed at the community in order to improve its overall ownership of EPI activities.

A quantitative study to assess factors associated with the vaccine status of zero-dose children in the community is needed to develop sustainable strategies that promote vaccine acceptance and overcome contextual barriers.

## References

[1] OMS. Plan d’Action Mondial pour les Vaccins 2011–2020 [Global vaccine action plan 2011–2020]. Genève: Bibliothèque de l’OMS, 2011 [cited n.d.]; p. 1–77. Available from: https://www.afro.who.int/sites/default/files/2017-06/French.pdf

[2] Comité régional de l’Afrique. Renforcement de la protection et de la résilience des communautés: Stratégie régionale pour la participation communautaire (2023–2030) dans la Région africaine de l’OMS (Document AFR/RC73/9) [Strengthening community protection and resilience: Regional strategy for community engagement (2023–2030) in the WHO African Region (Document AFR/RC73/9)]. Gaborone: Organisation Mondiale de la Santé, Bureau Régional de l’Afrique, 2023; p. 2. Rapport No. AFR/RC73/R3.

[3] Yao GHA, Aka LBN, Manouan NJM, et al. Knowledge, and attitudes of civil society organizations in the implementation of the expanded program on immunization in Côte D’ivoire. Santé Publique. 2014;26(1):99–106. 10.3917/spub.137.0099 24893521

[4] Chaney SC, Benjamin P, Mechael P. Déterminer l’élément important parmi de nombreuses informations: une revue documentaire portant sur l’utilisation efficace de l’écoute sociale numérique pour la promotion de la vaccination [Finding the signal through the noise: A landscape review of effective use of digital social listening for immunisation demand generation]. Westlake, Cape Town: HealthEnabled; 2021.

[5] Muhoza P, Danovaro-Holliday MC, Diallo MS, et al. Routine vaccination coverage – Worldwide, 2020. MMWR Morb Mortal Wkly Rep. 2021;70(43):1495–1500. https://doi.org/10.15585/mmwr.mm7043a1. Erratum in: MMWR Morb Mortal Wkly Rep. 2021;70(46):1620. https://doi.org/10.15585/mmwr.mm7046a7 10.15585/mmwr.mm7043a1 PMC8553029 34710074

[6] OMS. Projet de treizième programme général de travail, 2019–2023 (A71/4) [Draft thirteenth general programme of work, 2019–2023 (A71/4)]. Genève: Organisation Mondiale de la Santé, 2018 [cited n.d.]; p. 50. Available from: https://apps.who.int/gb/ebwha/pdf_files/WHA71/A71_4-fr.pdf

[7] World Health Organization. Fulfilling a promise: Ensuring immunization for all in Africa. Brazzaville: WHO Regional Office for Africa; 2016.

[8] United Nations Children’s Fund (UNICEF). The state of the world’s children 2023: For every child, vaccination. Executive summary. Florence: UNICEF Innocenti – Global Office of Research and Foresight; 2023 [cited n.d.]. Available from: https://www.unicef.org/media/108161/file/SOWC-2023-full-report-English.pdf

[9] Ministère de la Santé, Togo. Politique nationale de santé [National health policy]. Lomé, Togo: Ministère de la Santé; 2010.

[10] Ministère de la Santé, de l’Hygiène Publique et de l’Accès Universel aux Soins. Annuaire des statistiques sanitaires, année 2021, Togo [Health statistics yearbook, 2021, Togo]. Lomé: Inseed.tg, 2021; p. 211.

[11] Ministère de la Santé, de l’Hygiène Publique et de l’Accès Universel aux Soins. Analyse de la situation du Programme élargi de vaccination dans le cadre de l’élaboration de la Stratégie nationale de vaccination Togo [Analysis of the expanded programme on immunization situation in the context of developing Togo’s National Vaccination Strategy]. Lomé, Togo: Ministère de la Santé, de l’Hygiène Publique et de l’Accès Universel aux Soins, 2022; p. 28.

[12] Djanda L. Évaluation de la mise en œuvre de l’approche vaccinale « Atteindre chaque district » dans le district sanitaire de Tandjoaré au Togo en 2018 [Evaluation of the implementation of the ‘Reach Every District’ vaccination approach in the Tandjoaré health district, Togo, in 2018] [homepage on the Internet]. Alexandrie: Université Senghor; 2018 [cited 2023 Dec 04]. Available from: https://dicames.online/jspui/bitstream/20.500.12177/5327/1/DjandaLorimpo.pdf

[13] Ekouevi DK, Gbeasor-Komlanvi FA, Yaya I, et al. Incomplete immunization among children aged 12–23 months in Togo: A multilevel analysis of individual and contextual factors. BMC Public Health. 2018;18(1):952. 10.1186/s12889-018-5881-z PMC6090752 30071824

[14] Ministère de la Santé et de la Protection Sociale. Plan national de développement sanitaire 2017–2022 [National Health Development Plan 2017–2022]. Version définitive. Togo: Ministère de la Santé et de la Protection Sociale; 2017.

[15] Ministère de la Santé et de la Protection Sociale. Plan pluriannuel complet 2016–2020 du programme élargi de vaccination (Togo) [Comprehensive multi-year plan 2016–2020 for the expanded programme on immunization (Togo)]. Lomé: World Health Organization, 2015; p. 95.

[16] Ministère de la Santé et de la Protection Sociale du Togo. Plan intégré de communication du programme élargi de vaccination Togo (2018–2020) [Integrated communication plan for the expanded programme on immunization in Togo (2018–2020)]. Lomé: Ministère de la Santé et de la Protection Sociale du Togo, 2018; p. 1–77.

[17] Ministère de la Santé, de l’Hygiène Publique et de l’Accès Universel aux Soins. Stratégie nationale de vaccination 2023–2026 (Togo) [National vaccination strategy 2023–2026 {Togo}]. Lomé: Ministère de la Santé, de l’Hygiène Publique et de l’Accès Universel aux Soins, 2022; p. 1–37.

[18] Assemblée Nationale (Togo). Loi portant Code de la santé publique de la République togolaise, 2009 [Law establishing the public health code of the Togolese Republic (2009)]. Lomé: Population Reference Bureau, 2009; p. 1–114.

[19] Isabeaux KM, Lior M, Leah E, Maria F, Sow MK, Gbeasor-Komlanvi F. Points à retenir du dialogue virtuel de l’Accélérateur entre le Togo et la Guinée sur la santé communautaire et l’intégration de la vaccination [Key takeaways from the accelerator’s virtual dialogue between Togo and Guinea on community health and vaccination integration]. Lomé: Health Systems Strengthening Accelerator; 2022.

[20] Bell J, Lartey B, Spickernell G, et al. Applying a social-ecological model to understand factors impacting demand for childhood vaccinations in Nigeria, Uganda, and Guinea. SSM Qual Res Health. 2022;2:100180. 10.1016/j.ssmqr.2022.100180 PMC9748306 36531294

[21] Olaniyan A, Isiguzo C, Agbomeji S, Akinlade-Omeni O, Ifie B, Hawk M. Barriers, facilitators, and recommendations for childhood immunisation in Nigeria: Perspectives from caregivers, community leaders, and healthcare workers. Pan Afr Med J. 2022;43:97. 10.11604/pamj.2022.43.97.35797 PMC9816885 36660086

[22] Nguenga Kamani NL, Mankollo Bassong OY, Tchinda Fossi C, Nkoum Benjamin Alexandre N. Facteurs associés à la Non Observance du Calendrier Vaccinal des Enfants de 0 à 11 mois dans le District de Santé de Djoungolo - Cameroun [Factors associated with non-adherence to the vaccination schedule for children aged 0–11 months in the Djoungolo health district – Cameroon]. Health Sci Dis. 2020 [cited n.d.];21(2):111–117. Available from: https://www.hsd-fmsb.org/index.php/hsd/article/view/1827

[23] Barro M, Nombré T, Sanogo B, Ouattara ABI, Cessouma R, Nacro B. Les occasions manquees de vaccination en milieu hospitalier pediatrique de Bobo-Dioulasso (Burkina Faso) [Missed opportunities for immunization in pediatric unit of the Bobo-Dioulasso hospital (Burkina Faso)]. Mali Med. 2016 [cited n.d.];31(4):30–36. Available from: https://www.bibliosante.ml/handle/123456789/3147 30079653

[24] UNICEF. Fiche d’information Togo, Analyse des données pour l’apprentissage et l’équité utilisant les données MICS [Togo factsheet: Analysis of data for learning and equity using MICS data]. Lomé: UNICEF, 2021; p. 1–40.

[25] Ouédraogo LT, Ouédraogo SM, Ouédraogo ZT, et al. Déterminants du non-respect du calendrier vaccinal du programme élargi de vaccination au niveau district sanitaire: Cas du district sanitaire de Boussé, Burkina Faso [Factors for non-observance of the extended program timetable for vaccination in health districts: The case of Boussé in Burkina Faso]. Med Mal Infect. 2006;36(3):138–143. French. 10.1016/j.medmal.2006.01.005 16581215

[26] Traore K, Kone B, Sylla C, et al. Knowledge, attitudes and practices of tetanus vaccination by women of childbearing age in the health district of Commune I of Bamako (Mali). Greenfort Int J Appl Med Sci. 2024;2(5):179–186. 10.62046/gijams.2024.v02.i05.002

[27] Dutta T, Agley J, Meyerson BE, Barnes PA, Sherwood-Laughlin C, Nicholson-Crotty J. Perceived enablers and barriers of community engagement for vaccination in India: Using socioecological analysis. PLoS One. 2021;16(6):e0253318. 10.1371/journal.pone.0253318 PMC8232440 34170920

[28] Yawn BP, Xia Z, Edmonson L, Jacobson RM, Jacobsen SJ. Barriers to immunization in a relatively affluent community. J Am Board Fam Pract. 2000;13(5):325–332. 11001002

[29] World Health Organization. Implementation guide for vaccination of health workers. Geneva: World Health Organization; 2022.

[30] Boko AK. Étude des causes du non-respect du calendrier vaccinal des enfants de 0 à 11 mois dans le district sanitaire de Danyi [Study of the causes of non-compliance with the vaccination schedule for children aged 0 to 11 months in the Danyi health district]. Ouidah-Bénin: Université d’Abomey-Calavi; 2008.

[31] The UN Refugee Agency (UNHCR). Norme de dotation en personnel pour les soins de santé primaires [Primary health care staffing standard]. In: Emergency handbook. Geneva, Switzerland: United Nations High Commissioner for Refugees (UNHCR); 2024.

[32] Djimet S. Les barrières à la demande de service de vaccination chez les populations nomades de Danamadji, Tchad [Barriers to the demand for vaccination services among nomadic populations in Danamadji, Chad]. Leiden: African Studies Centre Leiden; 2017.

[33] Organisation Mondiale de la Santé. Suivi des inégalités en matière de vaccination: Manuel pratique [Inequality monitoring in immunization: A step-by-step manual]. Genève: World Health Organization, 2020; p. 87.

[34] Yaya BFIK, Kouanda S, Hinson L, Collymore Y, Ba-Nguz A, Bingham A. Perceptions des communautés sur le paludisme et les vaccins: Étude qualitative réalisée dans les districts sanitaires de Kaya et Houndé, au Burkina Faso [Community perceptions of malaria and vaccines: A qualitative study conducted in the Kaya and Houndé health districts, Burkina Faso]. Glob Health Promot. 2014;21(1):76–87. 10.1177/1757975913507729 24496777

[35] Alabadi M, Pitt V, Aldawood Z. A qualitative analysis of social-ecological factors shaping childhood immunisation hesitancy and delay in the eastern province of Saudi Arabia. Vaccines. 2023;11(9):1400. 10.3390/vaccines11091400 PMC10536341 37766077

[36] Ekezie W, Awwad S, Krauchenberg A, et al. Access to vaccination among disadvantaged, isolated and difficult-to-reach communities in the WHO European Region: A systematic review. Vaccines. 2022;10(7):1038. 10.3390/vaccines10071038 PMC9324407 35891201

[37] Sutovskaya DV, Pyzhyanova PA, Gabdullina EV, Makunts AA, Kuzmenko AV. Adherence to vaccination of health workers and populations. Russ Pediatr J. 2023;26(3):205–211. 10.46563/1560-9561-2023-26-3-205-211

[38] Topuzoglu A, Ay P, Hidiroglu S, Gurbuz Y. The barriers against childhood immunizations: A qualitative research among socio-economically disadvantaged mothers. Eur J Public Health. 2007;17(4):348–352. 10.1093/eurpub/ckl250 17090559

[39] Dhaliwal BK, Chandrashekhar R, Rattani A, et al. Community perceptions of vaccination among influential stakeholders: Qualitative research in rural India. BMC Public Health. 2021;21(1):2122. 10.1186/s12889-021-12188-4 PMC8600485 34794415

[40] Aslam F, Babar ZUD, Madni A, Asghar M, Yue Y. Unveiling and addressing implementation barriers of vaccination communication strategy: Perspectives from government officials at national and provincial levels. Hum Vaccin Immunother. 2022;18(7):2153513. 10.1080/21645515.2022.2153513 PMC9766463 36494089

[41] Bile AS, Ali-Salad MA, Mahmoud AJ, et al. Assessing vaccination delivery strategies for zero-dose and under-immunized children in the fragile context of Somalia. Vaccines. 2024;12(2):154. 10.3390/vaccines12020154 PMC10892412 38400137

[42] Mahmud S. Citizen participation in the health sector in rural Bangladesh: Perceptions and reality. IDS Bull. 2004;35(2):11–18. 10.1111/j.1759-5436.2004.tb00116.x

[43] Banque mondiale. Investir dans les pauvres et les vulnérables pour une prospérité inclusive: Une évaluation de la pauvreté et de la situation du genre au Togo en juin 2022 [Investing in the poor and vulnerable for inclusive prosperity: An assessment of poverty and the gender situation in Togo, June 2022] [rapport exécutif]. Washington, DC: Banque mondiale, 2022; p. 88.

[44] Banque mondiale. Investir dans les pauvres et les vulnérables pour une prospérité inclusive: une évaluation de la pauvreté et de la situation du genre au Togo [World Bank Group. Investing in the Poor and Vulnerable for an Inclusive Prosperity: Poverty and Gender Assessment in Togo]. [rapport exécutif] [homepage on the internet] Washington, DC: World Bank Group; 2022 [cited 2023 Feb 25]. Available from: http://documents.worldbank.org/curated/en/099110623151041764

[45] Ministère de la Santé, de l’Hygiène Publique et de l’Accès Universel aux Soins, Togo. Plan stratégique national de lutte intégrée contre les MTN 2022–2023 au Togo [National Strategic Plan for the Integrated Control of Neglected Tropical Diseases (NTDs) 2022–2023 in Togo] [plan stratégique]. Lomé: World Health Organization, 2022; p. 82.

